# Adaptive and Maladaptive Perfectionism in Relation to Orthorexic Behaviors: The Mediating Role of Self-Compassion Dimensions

**DOI:** 10.3390/jcm14238288

**Published:** 2025-11-21

**Authors:** Adrianna Solska, Joanna Furmańska, Małgorzata Szcześniak

**Affiliations:** 1 Maria Skłodowska-Curie Elementary School No. 3 with Bilingual Sections, 72-010 Police, Poland; 2 Institute of Psychology, University of Szczecin, 71-017 Szczecin, Poland

**Keywords:** orthorexia, adaptive perfectionism, maladaptive perfectionism, self-compassion

## Abstract

**Background/Objectives**: An excessive focus on the consumption of “healthy” or “clean” foods may develop into an obsessive pattern of eating behavior. Orthorexia, a relatively recent construct within the field of eating and feeding disorders, reflects this maladaptive preoccupation with dietary purity. Although orthorexia has received increasing empirical attention, its etiological pathways and maintaining mechanisms remain insufficiently understood. Previous research has identified perfectionism as a potential risk factor contributing to the development and persistence of orthorexic tendencies. Accordingly, the present study aimed to examine the association between perfectionism and orthorexic behaviors and to explore the mediating role of self-compassion, conceptualized as the capacity to respond to personal suffering with kindness, mindfulness, and a sense of shared humanity, in this relationship. **Methods**: The study sample consisted of 227 participants aged 18 to 55 years. Measures included a self-designed sociodemographic questionnaire, the Bratman Orthorexia Test (BOT), the ORTO-15, the Adaptive and Maladaptive Perfectionism Questionnaire (AMPQ), and the Self-Compassion Scale (SCS-PL). **Results**: Findings confirmed a positive association between maladaptive perfectionism and orthorexic behaviors, as well as relationships between both adaptive and maladaptive perfectionism and various dimensions of self-compassion. Self-compassion and its dimensions were also found to mediate the relationship between perfectionism and orthorexic behaviors. **Conclusions**: The study highlights the importance of both perfectionism and self-compassion as factors associated with orthorexic behaviors. Given that self-compassion is linked to mental health and may serve as a buffer against self-critical cognitive processes, its assessment is particularly relevant, especially as research suggests it is a skill that can be cultivated.

## 1. Introduction

The so-called healthy lifestyle is intended to promote well-being and longevity. However, an excessive focus on healthy eating can become obsessive and take on a pathological character, resulting in negative consequences [1,2,3]. In recent years, an increasing number of studies have focused on maladaptive eating behaviors, also highlighting a rise in the prevalence of eating disorders [4,5] and recognizing that disordered eating behaviors have become a public health concern [6]. These behaviors carry implications for overall health, as well as psychological, social, and physical well-being. Consequently, it is unsurprising that comprehensive research into the factors contributing to the development and maintenance of these behaviors is required [6]. One relatively recent example of an obsessive focus on “healthy” eating and “correct” nutrition is orthorexia [7], which is characterized by the pursuit of a “perfect diet” [8].

### 1.1. Orthorexia—Characteristics and Risk Factors

Orthorexia, first described by Bratman in 1997, is characterized by an obsessive and excessive focus on exclusively healthy eating [9]. Individuals exhibiting orthorexic behaviors focus on the quality and composition of food rather than on achieving a desired body shape [9]. Orthorexic behaviors include following a strict diet, obsessively avoiding artificial colors, preservatives, flavorings, food produced non-ecologically, or containing pesticides, contaminants, and other highly processed substances [7]. As a result, individuals may devote excessive time to meal planning and physical activity, which can lead to social withdrawal, impaired family relationships, or even neglect of professional responsibilities [10].

Horovitz and Argyrides [6] note that due to the lack of clear diagnostic criteria, it is difficult to distinguish precisely between orthorexia, defined as an obsessive fixation on “clean” and “healthy” food [11], and orthorexia nervosa, which represents a more severe and clinically significant form of this obsession, associated with intense anxiety and disruptions in daily functioning resulting from extreme dietary restrictions [7].

Based on an extensive literature review, Dunn and Bratman [12] proposed the following diagnostic criteria for orthorexia: (1) fundamental characteristics—compulsive behavior and/or mental preoccupation regarding dietary practices that are believed to promote optimal health; (2) emotional and physical responses to dietary transgression—exaggerated fear of illness, personal impurity, and/or negative physical sensations, accompanied by fear and shame; (3) escalation from disordered eating to pathology—progressively more restrictive dietary practices involving increasingly frequent and/or severe “cleanses” or partial fasts. They further highlight additional factors that may be considered for diagnostic purposes.

Imposing strict dietary rules on oneself and adhering to them meticulously may provide an apparent sense of control [13], as well as feelings of self-fulfillment and superiority over others, and may even alleviate anxiety about potential illness development [14]. Deviations from the prescribed diet can lead to intense frustration, with each failure provoking guilt and even self-disgust. This, in turn, may result in a compulsion to punish oneself by increasing dietary rigidity, and in extreme cases, resorting to fasting [15].

Among the risk factors contributing to the development and maintenance of orthorexic behaviors, psychological and socio-cultural influences have also been highlighted. Based on a literature review, McComb and Mills [16] indicated that a higher prevalence of orthorexia was positively associated with obsessive-compulsive traits, psychopathology, disordered eating, a history of eating disorders, dieting, poor body image, drive for thinness, and perfectionism. High levels of stress or low satisfaction with various aspects of one’s life may prompt individuals to turn to food as a means of regaining an illusion of control [17,18]. At the socio-cultural level, idealized images of health and “clean eating” can exert pressure, while adherence to rigid dietary rules may provide a sense of purpose, self-placement, and conformity to community standards oriented toward promoting and maintaining health [19,20]. However, these psychological, socio-cultural, and certain lifestyle factors (e.g., competitive sports participation or specific dietary habits) require further research to clarify their role in the etiology and persistence of orthorexic behaviors [21].

In response to the need for a more in-depth analysis of factors associated with orthorexic behaviors, perfectionism has drawn attention at the individual level. As a personality trait, it is associated with setting excessively high standards and engaging in self-critical evaluation [22]. Consequently, perfectionism can be examined as one of the transdiagnostic risk factors for eating disorders [23,24].

### 1.2. Adaptive and Maladaptive Perfectionism

For individuals with perfectionistic tendencies, merely performing a task adequately is insufficient [25]. They strive for an ideal and self-improvement, yet often feel they could have done better. High self-criticism, striving for perfection, and setting elevated personal standards are defining features of perfectionism [22]. Cattell [26] described perfectionism as a core personality factor that correlates with neuroticism, which becomes apparent when an individual loses control or encounters failure [27]. This manifests in difficult situations as fear of negative evaluation and a need for strong social approval [28]. Individuals with perfectionistic tendencies are particularly sensitive to evaluation and, consequently, seek social recognition and approval [29]. Signs of perfectionism are visible from childhood and adolescence [30], and its influence can be observed across almost all areas of functioning [31], although in most cases, its effects are domain-specific [32].

The literature describes multidimensional models of perfectionism [22,25,33]. Of particular interest is a two-dimensional construct that distinguishes between positive and negative perfectionism [34]. Adaptive perfectionism is considered healthy and is characterized by striving for excellence, a flexible approach to personal standards, and acceptance of outcomes regardless of results. In this case, increasing the demands on oneself fosters self-development and satisfaction, while challenges are accepted [34]. Maladaptive perfectionism, on the other hand, is driven by a fear of making mistakes. Individuals with this type of perfectionism experience societal pressure to be perfect, which leads them to set unrealistically high standards they cannot meet [35]. The primary predictor of maladaptive perfectionism is a fear of failure and negative evaluation. Such individuals set unrealistic goals because they believe that achieving them will earn social approval [36].

Both adaptive and maladaptive perfectionism significantly influence an individual’s life, including health outcomes. Adaptive perfectionism is associated with higher engagement in preventive health behaviors, whereas maladaptive perfectionism is linked to lower engagement in preventive behaviors and higher psychological stress [37], and can pose a risk for the development of psychopathologies [38,39]. Perfectionism is also recognized as a risk factor for eating disorders [25,40,41].

Although adaptive perfectionism can be beneficial for achieving challenging goals and continuous self-improvement, maladaptive perfectionism more frequently leads to stress and potential negative consequences due to unrealistic standards and rigorous self-criticism. Consequently, developing self-compassion may help individuals cope with the destructive aspects of perfectionism, enabling a gentler approach to personal imperfections [42]. Following this rationale, the present study focuses on examining the relationship between perfectionism and orthorexic behaviors, with self-compassion as a potential mediating factor.

### 1.3. Self-Compassion

Neff [43] defined the construct of self-compassion as sensitivity to one’s own suffering, coupled with care and understanding, as well as the recognition and acceptance of one’s imperfections. This approach to the self stands in contrast to selfishness, egocentrism, harshness, and narcissism [44], and differs from self-esteem, which can also negatively impact well-being, foster feelings of superiority, and lead to self-absorption; high self-esteem scores have been found to correlate with narcissism [45].

Self-compassion involves approaching oneself with kindness and understanding, without engaging in social comparison as is typical for individuals with high self-esteem [46]. Self-compassionate individuals do not harshly criticize their mistakes, though this is not equivalent to self-indulgence [47]. The focus is not on rejecting difficult emotions, but rather on being aware of them, regulating them, and accepting one’s imperfections. In this sense, an excessive focus on failure can hinder learning from mistakes and lower self-worth, whereas maintaining a balanced emotional perspective without suppressing painful feelings characterizes self-compassion [43].

Individuals who exhibit self-compassion demonstrate better emotion regulation, which allows them to acknowledge and accept challenging health diagnoses rather than becoming overwhelmed by negative judgments and emotions [48]. Self-compassion has been associated with health-related outcomes such as lower perceived stress [49,50], attenuated physiological stress responses [51], and engagement in health-promoting behaviors [52]. Consequently, practicing self-compassion supports a healthy lifestyle, leading to fewer physical symptoms and enhanced overall well-being [53].

### 1.4. Self-Compassion as a Mediator

The current literature review indicates a significant association between perfectionism and orthorexic traits [54,55]. Excessive concern for health may contribute to the development of orthorexia, as perfectionists strive for the ideal of health by strictly adhering to self-imposed, elevated dietary standards [56]. Consequently, a perfectionistic approach to health, combined with anxiety about not achieving perfect health, may serve as a predictor of orthorexic behaviors [57]. Orthorexia can thus be viewed as a manifestation of perfectionism in the domain of health, in which food functions as a highly controlled and regimented element of life. Elevated perfectionistic traits may predict that orthorexic symptoms will intensify over time, as the need for control and desire for continuous improvement increase [21].

Oberle et al. [58] noted a link between orthorexia and high levels of maladaptive perfectionism. However, Michalska and colleagues [59], analyzing data from 607 participants, reported different findings. Their study suggested that while perfectionism correlates with orthorexic behaviors, higher levels of both adaptive and maladaptive perfectionism were associated with eating behaviors closer to normative patterns. These ambiguous results indicate that mediating factors may play a crucial role in this relationship.

Moreover, individuals with high levels of perfectionism may struggle to cultivate self-compassion [60], and the obsessive concern with avoiding mistakes characteristic of maladaptive perfectionism may negatively affect the capacity for self-kindness [61]. Research also indicates that practicing self-compassion can help individuals with maladaptive perfectionism reduce depressive symptoms or even prevent their onset [62,63,64,65,66]. Furthermore, perfectionists who exhibit self-compassion report higher perceived quality of life and lower intensity of symptoms such as excessive focus on potential mistakes or the need for approval [67].

Thus, self-compassion may act as a buffer, mitigating the negative effects of perfectionism and fostering a more balanced and healthier approach to daily life. Conversely, when self-criticism predominates over self-compassion, individuals may focus on perceived negative aspects of their character or appearance and subsequently set unrealistically high standards for themselves in pursuit of these ideals.

### 1.5. Aim of the Study

Based on a literature review and the existing gap in knowledge regarding the mediating role of self-compassion in the relationship between perfectionism and orthorexic behaviors, the primary aims of the present study were to examine: (1) the relationship between adaptive and maladaptive perfectionism and orthorexic behaviors; and (2) the role of self-compassion and its dimensions as potential mediators in this relationship.

The study sought to answer the following research questions: (1) Is there a relationship between adaptive and maladaptive perfectionism and orthorexic behaviors in a sample of adults? (2) Is there a relationship between adaptive and maladaptive perfectionism and self-compassion/its dimensions? (3) Do self-compassion/its dimensions serve as mediating factors between adaptive and maladaptive perfectionism and orthorexic behaviors?

The following hypotheses were formulated:

**H1.**  *Higher levels of maladaptive and adaptive perfectionism will be associated with higher levels of orthorexic behaviors*.

**H2.**  *Orthorexic behaviors will be positively associated with self-judgment, isolation, and over-identification and negatively associated with self-kindness, common humanity, mindfulness, and self-compassion overall.*

**H3.**  *Higher levels of maladaptive perfectionism will be associated with higher self-judgment, isolation, and over-identification, and lower levels of self-kindness, common humanity, mindfulness, and self-compassion overall*.

**H4.**  *Higher levels of adaptive perfectionism will be associated with higher self-kindness, common humanity, mindfulness, and self-compassion overall, and lower self-judgment, isolation, and over-identification*.

**H5.**  *Self-compassion overall and its dimensions will mediate the relationship between maladaptive/adaptive perfectionism and orthorexic behaviors*.

## 2. Materials and Methods

### 2.1. Participants and Procedure

The sample consisted of 227 respondents aged 18 to 55 years (*M* = 25.49; *SD* = 5.44). Women comprised 77.5% of the sample (*n* = 176). The majority of participants had a higher education degree (45.8%) and were currently pursuing further education (42.7%). Over 60% of respondents reported being in an ongoing romantic relationship. Regarding health-related variables, nearly half of the participants (47.1%) indicated that they did not engage in regular physical activity, while slightly more than half (52.9%) reported participating in sports. Concerning eating habits, the vast majority of participants (83.9%) stated that they did not count calories in their daily meals. The mean BMI was *M* = 22.62 (*SD* = 3.61), ranging from 15.79 to 38.52. Table 1 presents the sociodemographic characteristics of the study participants.

The study was designed to be anonymous and voluntary, with no financial compensation provided to participants. Data were collected using an online survey. Prior to completing the survey, participants were required to provide informed consent to participate in the study. They then proceeded to the first part of the survey, which collected sociodemographic information, followed by the second part consisting of selected questionnaires.

A purposive sampling method was employed, using a snowball technique and excluding individuals under 18 years of age. To recruit the study sample, advertisements were posted in specialized Facebook groups related to nutrition, as well as on other profiles and websites focused on healthy lifestyles. The title and description of the study informed potential participants about its topic, allowing them to choose whether to participate. Each question in the survey was marked as required to ensure responses were provided for all items.

### 2.2. Bratman Orthorexia Test

The Bratman Orthorexia Test (BOT) is one of the oldest self-assessment tools for orthorexia. It consists of 10 items, which respondents answer with “YES” or “NO.” However, the test lacks established psychometric properties [12] and is primarily used in screening studies. Given its use in several studies [68,69] and its screening purpose, the BOT was employed alongside a second tool for assessing orthorexic behaviors (ORTO-15) solely to complement the description of results. In the present study, Cronbach’s alpha for the BOT was 0.61.

### 2.3. ORTO-15

The ORTO-15 questionnaire is designed for the preliminary self-assessment of potential risk for orthorexia, defined as an obsessive concern with healthy eating that may lead to dietary restrictions and health problems [70,71]. The tool was developed based on the BOT and assesses the severity of orthorexic behaviors, encompassing clinical symptoms as well as cognitive and emotional aspects of functioning in individuals with orthorexia [72]. Participants responded to 15 items using a 4-point Likert scale. In the present study, Cronbach’s alpha for the ORTO-15 was 0.68. In the present study, the correlation between the questionnaires measuring orthorexic behaviors indicated a moderate positive relationship between the BOT and the ORTO-15 (*r* = 0.50, *p* < 0.001).

### 2.4. Adaptive and Maladaptive Perfectionism Questionnaire (AMPQ)

The Adaptive and Maladaptive Perfectionism Questionnaire (AMPQ) is designed to assess levels of adaptive perfectionism (positive, characterized by motivation for self-improvement, high personal standards, and satisfaction with achievements) and maladaptive perfectionism (negative, characterized by excessive self-criticism, unrealistic expectations, and fear of making mistakes). The questionnaire consists of 35 items, which participants respond to using a 5-point Likert scale [34]. In the present study, Cronbach’s alpha reliability was 0.95 for maladaptive perfectionism and 0.91 for adaptive perfectionism.

### 2.5. Self-Compassion Scale (SCS)

The Self-Compassion Scale (SCS) is designed to assess six dimensions of self-compassion: (1) self-kindness, (2) self-judgment, (3) common humanity, (4) isolation, (5) mindfulness, and 6) over-identification. Participants responded to 26 items using a 5-point Likert scale [43]. In the present study, Cronbach’s alpha reliabilities for the subscales were: self-kindness = 0.87, self-judgment = 0.76, common humanity = 0.73, isolation = 0.71, mindfulness = 0.74, and over-identification = 0.74. According to Neff [43], self-compassion may also be conceptualized as a single higher-order construct. In the present study, the reliability of the overall self-compassion score was excellent, with a Cronbach’s alpha of α = 0.92.

### 2.6. Statistical Analysis

Statistical analyses were conducted using the Statistical Package for the Social Sciences (SPSS), version 25. To assess the distribution of variables, the criterion proposed by Tabachnick and Fidell [73] was used, according to which skewness and kurtosis values within the range of ±2 indicate an approximately normal distribution. After verifying this criterion, the assumption of approximate normality was accepted, and Pearson’s *r* correlation was applied. Independent samples *t*-tests were employed to compare groups, along with Levene’s test to assess the equality of variances for each variable. Mediation analyses were performed using the PROCESS macro for SPSS (Model 4) developed by Hayes [74]. The mediation analysis was conducted using a bootstrapping procedure with 5000 resamples and 95% confidence intervals (CIs). A mediating effect was deemed significant if the confidence interval did not encompass zero.

In addition to assessing the statistical significance of the indirect effects, the effect size (v^2^) for the model was calculated using the Stats Tools Package. Following the guidelines of Gaskin et al. [75], thresholds of 0.01, 0.075, and 0.175 were applied to indicate small, medium, and large effects, respectively.

## 3. Results

### 3.1. Descriptive Statistics

In the first step, basic descriptive statistics and normality indices for the study variables were computed, as presented in Table 2. The values of skewness and kurtosis for all variables ranged between −0.70 and 1.10, which falls within the acceptable range of ±2, indicating approximate normality [73]. Therefore, the assumption of normality was considered met, and Pearson’s *r* correlations were applied in subsequent analyses.

### 3.2. Relationship Between Orthorexic Behaviors, Perfectionism and Self-Compassion Overall/Its Dimensions

In accordance with the adopted research model, the relationship between adaptive and maladaptive perfectionism and orthorexic behaviors was first examined. The results indicated a significant positive association between maladaptive perfectionism and orthorexic behaviors. Significant relationship was also found between adaptive perfectionism and orthorexic behaviors, as measured by the BOT questionnaire. Thus, the first hypothesis (H1) was confirmed. Detailed results are presented in Table 3.

The findings also offered strong support for hypothesis H2, revealing that orthorexic behaviors were negatively related to self-kindness, mindfulness, and overall self-compassion, while demonstrating positive associations with self-judgment, isolation, and over-identification across both assessment tools. Similarly, isolation and over-identification were positively related to orthorexia. In contrast, self-compassion, self-kindness and mindfulness, were negatively associated with orthorexic behaviors. The strongest negative correlation was observed between overall self-compassion and the ORTO-15. Common humanity was the only variable that showed no significant correlation with either the ORTO-15 or the BOT. Overall, the results indicate that orthorexic behaviors are linked to higher levels of self-critical dimensions of self-compassion and lower levels of self-kindness, mindfulness, and overall self-compassion.

### 3.3. Relationship Between Perfectionism and Self-Compassion

In the next step, the relationship between maladaptive/adaptive perfectionism and the individual dimensions of self-compassion was examined. The results revealed significant positive associations between maladaptive perfectionism and self-judgment, isolation, and over-identification. Simultaneously, significant negative correlations were observed with self-kindness, common humanity, mindfulness, and self-compassion overall, thereby confirming the third (H3) hypothesis. A significant positive correlation was also found between adaptive perfectionism and mindfulness, common humanity, and self-judgment (see Table 4), partially supporting the fourth (H4) hypothesis. This pattern of results suggests that individuals with maladaptive perfectionistic tendencies may be more susceptible to excessive self-judgment, social withdrawal, and heightened engagement with distressing thoughts and emotions. Moreover, they appear to exhibit lower levels of self-kindness, reduced acceptance of imperfections and mistakes as natural aspects of the human experience, and a diminished capacity to observe their thoughts and emotions with non-judgmental awareness.

### 3.4. Self-Compassion as a Mediator Between Perfectionism and Orthorexic Behaviors

Table 5 presents the results of the mediation analyses examining the indirect effects of perfectionism dimensions on orthorexic behaviors through self-kindness, self-judgment, common humanity, isolation, mindfulness, over-identification, and self-compassion overall. These findings partially confirm the fifth hypothesis (H5) and indicate that both maladaptive and adaptive perfectionism may exert indirect effects on orthorexic behaviors, albeit through different mediational pathways.

Specifically, for maladaptive perfectionism, the indirect effects on orthorexic behaviors were significant for four mediators: self-judgment, isolation, over-identification, and overall self-compassion. In contrast, the positive dimensions of self-compassion (self-kindness, common humanity, and mindfulness) did not significantly mediate this relationship, as their confidence intervals included zero.

For adaptive perfectionism, significant mediation effects were observed only for self-judgment and mindfulness, but in opposite directions. Notably, mindfulness demonstrated a negative mediation effect, indicating that greater mindfulness associated with adaptive perfectionism functioned as a protective factor that mitigated orthorexic behaviors. No other self-compassion components significantly mediated this relationship.

Although seven mediation models were statistically significant and supported by bootstrapped confidence intervals (Table 5), all exhibited negligible effect sizes, suggesting limited practical relevance. Consequently, these findings should be interpreted with caution when considering their applied implications.

## 4. Discussion

The aim of the present study was to examine the direct relationship between adaptive and maladaptive perfectionism and orthorexic behaviors, and to investigate the mediating role of self-compassion and its dimensions on these associations. The results obtained generally support the proposed hypotheses.

With regard to the first hypothesis H1, it was confirmed. As expected, individuals with higher levels of both maladaptive and adaptive perfectionism tend to display greater intensity of orthorexic behaviors. These results align with previous research linking perfectionism to eating disorders [24,54]. This suggests that maladaptive perfectionism may represent an important risk factor in the development of a pathological preoccupation with healthy eating [76,77]. Moreover, the positive association between adaptive perfectionism, characterized by conscientiousness, achievement striving, and the pursuit of excellence, and orthorexia may reflect a coexistence with a tendency toward proper eating behaviors [59]. Finally, a systematic review and meta-analysis by Pratt et al. [21] demonstrated that both perfectionistic strivings and perfectionistic concerns are positively associated with orthorexia.

The findings also provided support for hypothesis H2. The results show that orthorexic behaviors are positively associated with self-judgment, isolation, and over-identification and are negatively associated with self-kindness, common humanity, mindfulness, and self-compassion overall. These results corroborate previous empirical findings. For example, recent study indicates that even minor lapses in a “healthy” diet can activate a cycle of self-judgment and corrective behaviors, intensifying rigid dietary control [13]. Hanna et al. [78] report that individuals who focus on food and are preoccupied with healthy eating are also more likely to experience social isolation. With respect to the relationship between orthorexia and the positive dimensions of self-compassion, prior work has consistently shown that orthorexia is negatively correlated with self-compassion and mindfulness [79,80]. Although research examining the self-kindness component of self-compassion in relation to orthorexic behaviors is limited, it is reasonable to assume that treating oneself with warmth, care, and understanding during times of perceived inadequacy may reduce eating-related behaviors characterized by rigid dietary rules.

Evidence from the study supported the third hypothesis H3, showing that higher maladaptive perfectionism was associated with higher levels of self-judgment, isolation, and over-identification, and with lower levels of self-kindness, common humanity, and mindfulness. Individuals with higher maladaptive perfectionism are more prone to self-criticism, feelings of isolation, and a tendency to over-identify with negative emotions. This is associated with a propensity for harsh self-judgment [81], experiencing feelings of isolation [82], and over-identification with personal difficulties [81]. At the same time, positive dimensions of self-compassion, such as self-kindness, mindfulness, and a sense of common humanity, were clearly reduced or absent in individuals with maladaptive perfectionism [61]. The absence of these positive psychological resources may increase the vulnerability to negative emotional states. According to the literature, cultivating positive self-compassion components in maladaptive perfectionists may serve a protective function, reducing the risk of anxiety and depressive symptoms [66,83].

Hypothesis H4 was partially confirmed. Positive correlations were observed between adaptive perfectionism and mindfulness, common humanity, and self-judgment. This suggests that individuals with higher adaptive perfectionism tend to be more mindful of their goals and to recognize the universality of human experiences. Consistently, Daniilidou [84] found that high personal standards, conceptually similar to adaptive perfectionism, were positively associated with self-kindness and mindfulness. Compared to maladaptive perfectionism, adaptive perfectionism is characterized by higher levels of positive self-compassion components and lower levels of negative aspects [66,85,86,87,88]. The presence of these positive resources may serve a protective function, reducing vulnerability to depressive symptoms [89]. Of particular interest is the positive correlation between adaptive perfectionism and self-judgment, which may appear counterintuitive. However, Linnett and Kibowski [61] noted that adaptive perfectionism is not solely linked to positive components; it can also co-occur with a tendency toward self-judgment. These somewhat ambiguous findings indicate that this relationship warrants further in-depth exploration. Notably, higher levels of self-judgment have also been associated with elevated personal expectations [86].

Finally, the findings provide support for the fifth hypothesis H5, particularly highlighting the mediating role of self-compassion and its specific dimensions in the association between maladaptive perfectionism and orthorexia. Our results suggest that maladaptive perfectionists who are prone to self-judgment, feelings of isolation, and over-identification with personal shortcomings, may assume rigid dietary preoccupations, characteristic of orthorexia. This points out to negative dimensions of self-compassion as potential targets for interventions aimed at reducing orthorexic behaviors in maladaptive perfectionistic individuals [66,88]. Kalika et al. [79] demonstrated that self-compassion is a key mediator in explaining the relationship between restrictive eating and orthorexia, indicating that low self-compassion leads to rigidity in dietary behaviors. Their later study [80] further suggested that self-compassion mediates the relationship between perfectionism and orthorexia, implying that low levels of self-compassion may increase the risk of developing eating disorders among perfectionists. Additionally, research shows that cultivating self-compassion skills can reduce the severity of orthorexic behaviors [89]. Conversely, higher self-compassion reinforces resilience against maladaptive perfectionistic behaviors, diminuishing the risk for orthorexia. Fulton [90] demonstrated that self-compassion can mediate between compassion toward others and mindfulness, suggesting that individuals who are more compassionate toward themselves cope better with personal and social pressures. The ability to treat oneself with kindness and acceptance is crucial for maintaining psychological and emotional health, particularly in the face of high expectations and stress.

For adaptive perfectionism in relation to orthorexia, our results revealed that only self-judgment and mindfulness among the self-compassion dimensions served a mediating function. This pattern suggest that even adaptive forms of perfectionism, typically associated with conscientiousness and goal-directed behavior [91], may still confer risk for orthorexic behaviors when accompanied by elevated self-criticism and reduced mindfulness. The mediating role of self-judgment indicates that individuals high in adaptive perfectionism may engage in harsh self-evaluation when their health-related standards are not perfectly achieved, thereby amplifying rigid and obsessive eating behaviors [68,92]. In contrast, mindfulness appears to exert a protective effect by fostering non-judgmental awareness and cognitive flexibility, which may mitigate the maladaptive consequences of perfectionistic striving [43,93].

In summary, the present study provides evidence for the association between perfectionism and orthorexic behaviors, as well as for the mediating role of self-compassion in this relationship. Given that self-compassion is associated with mental health and serves as a buffer against self-critical ruminative cognitive processes [44] and is linked to lower levels of psychopathology [94] and higher well-being [95], its assessment appears highly relevant. Importantly, self-compassion is not a fixed trait but a skill that can be developed [96] and applied to cope with mistakes, failure, and/or rejection [97]. Ferrari et al. [98], based on a meta-analysis, indicated that compassion-based interventions significantly improve eating habits, reduce rumination, and moderately impact psychopathology and mindfulness. Similarly, Krieger et al. [99] demonstrated in a randomized study assessing the effectiveness of a Mindful-Based Compassionate Living (MBCL) intervention that participants in the intervention group showed higher self-compassion scores compared to controls. Taylor et al. [100] reported significant increases in self-compassion from pre- to post-test in an intervention group, while Stutts [97] highlighted that multiple self-compassion interventions enhance self-compassion and improve psychological health. This topic contributes to understanding factors associated with orthorexic behaviors and encourages further research to better comprehend this emerging phenomenon.

## 5. Limitations and Future Directions

This study has several limitations. First, the cross-sectional correlational design limits the ability to draw conclusions about causality or directionality. While our findings suggest that perfectionism may contribute to disordered eating and that self-compassion could buffer this effect, these relationships cannot be conclusively established in the present study. Longitudinal and experimental studies are needed to elucidate the causal links between perfectionism, self-compassion, and orthorexic behaviors.

Second, although the mediation effects were statistically significant in four cases for maladaptive perfectionism (self-judgment, isolation, over-identification, and overall self-compassion) and in two cases for adaptive perfectionism (self-judgment and mindfulness), their magnitude was negligible, and interpretation should be approached with caution. This suggests that the mediators account only for a small portion of the variance, and that the relationship between these variables is likely more complex, with additional psychological mechanisms potentially contributing.

Third, the use of a purposive sample with snowball sampling limits the generalizability of the findings. The sample was convenient, and the online survey format excluded individuals without Internet access or those not using the platforms employed for data collection. Additionally, participants who opted to take part may have had a greater interest in the topic, potentially influencing the results. Future studies should aim to include underrepresented groups to enhance generalizability.

Demographically, the sample was predominantly composed of young women with higher education. Furthermore, the study did not examine the relationship between sociodemographic variables and orthorexic behaviors, which further limits the generalizability of the findings. Future research should aim to include more diverse populations and explore additional psychological factors that may influence the subjective assessment of orthorexic behaviors, such as social support and feelings of loneliness.

It is also important to note that no clinical verification of orthorexic behaviors was conducted. The study relied exclusively on self-report questionnaires, meaning that the results reflect participants’ subjective assessments rather than clinical diagnoses made by an interdisciplinary team. Moreover, some of the psychometric properties of the instruments used warrant further refinement [12,101,102].

Despite these limitations, the study advances current understanding by jointly examining adaptive and maladaptive perfectionism and exploring how specific self-compassion dimensions mediate their relationship with orthorexic behaviors. To the best of our knowledge, Kalika et al. [80] are the only authors who have tested self-compassion as a mediator between perfectionism and orthorexia, albeit using a different set of measurement instruments. No previous study has simultaneously contrasted adaptive and maladaptive perfectionism while modeling individual self-compassion dimensions (self-kindness, self-judgment, common humanity, isolation, mindfulness, over-identification) as mediators of orthorexic behaviors. The present study addresses this gap. These findings also have important implications for clinical practice, suggesting that interventions aimed at strengthening self-compassion may help reduce the impact of maladaptive and adaptive perfectionism on orthorexic behaviors.

## Figures and Tables

**Table 1:** Sociodemographic characteristics of the study sample (*N* = 227).

Sociodemographic Characteristics	
Respondents	*N* (%)
	227 (100%)
Sex	*N* (%)
Female	175 (77.5%)
Male	51 (22.2%)
Age (years)	*M* (*SD*); MIN–MAX
	25.49 (5.44); 18–55
Education	*N* (%)
Primary education	2 (0.9)
Lower secondary education	0 (0)
Vocational education	2 (0.9)
High school diploma	22 (9.7)
Currently pursuing higher education	97 (42.7)
Higher education	104 (45.8)
Relationship status	*N* (%)
In a relationship	138 (60.8)
Single	89 (39.2)
Physical activity	*N* (%)
Yes	120 (52.9)
No	107 (47.1)
Number of training days per week	*N* (%)
0	92 (40.5)
1–2	68 (30)
3–4	55 (24.2)
5–6	7 (3.1)
Every day	5 (2.2)
Calorie counting	*N* (%)
Yes	39 (17.2)
No	188 (82.8)
BMI (calculated based on participants’ height and weight)	*M* (*SD*); MIN–MAX
	22.62 (3.61) min: 15.79–max: 38.52

*N* = number of participants; % = percentage of responses; BMI = Body Mass Index.

**Table 2:** Descriptive statistics and normality tests for study variables (*N* = 227).

Variables	*M*	*SD*	Skewness	Kurtosis
ORTO-15	38.33	5.36	−0.27	1.10
BOT	3.12	1.98	0.40	−0.33
MP	64.08	20.22	−0.03	−0.56
AP	46.67	10.20	−0.57	0.24
Self-Kindness	15.20	4.54	−0.07	−0.54
Self-Judgment	15.67	4.29	−0.02	−0.63
Common Humanity	12.33	3.52	−0.15	−0.40
Isolation	12.60	4.10	−0.22	−0.70
Mindfulness	12.66	3.27	−0.01	−0.34
Over-Identification	13.37	3.57	−0.33	−0.23
Self-Compassion	76.56	17.72	0.029	0.050

BOT—The Bratman Orthorexia Test; MP—Maladaptive Perfectionism; AP—Adaptive Perfectionism.

**Table 3:** Values of Pearson’s r correlation coefficients between maladaptive/adaptive perfectionism, self-compassion/its dimensions and orthorexic behaviors (*N* = 227).

Variables	ORTO-15	Bratman Orthorexia Test
Maladaptive Perfectionism	0.37 ***	0.23 **
Adaptive Perfectionism	0.07	0.18 **
Self-Kindness	−0.25 ***	−0.08
Self-Judgment	0.45 ***	0.25 ***
Common Humanity	−0.05	0.04
Isolation	0.43 ***	0.21 **
Mindfulness	−0.14 *	−0.01
Over-Identification	0.38 ***	0.15 *
Self-Compassion	−0.39 ***	−0.16 *

* *p* < 0.05; ** *p* < 0.01; *** *p* < 0.001.

**Table 4:** Values of Pearson’s r correlation coefficients between maladaptive/adaptive perfectionism and dimensions of self-compassion (*N* = 227).

Self-Compassion	Maladaptive Perfectionism	Adaptive Perfectionism
Self-Kindness	−0.53 ***	0.052
Self-Judgment	0.72 ***	0.26 ***
Common Humanity	−0.31 ***	0.15 *
Isolation	0.72 ***	0.05
Mindfulness	−0.45 ***	0.18 **
Over-Identification	0.66 ***	0.09
Self-Compassion	−0.76 ***	−0.02

* p < 0.05; ** p < 0.01; *** p < 0.001.

**Table 5:** Self-compassion and its dimensions as mediators between maladaptive/adaptive perfectionism and orthorexia measured by the ORTO and the BOT (*N* = 227).

	a Path	b Path	c Path	c’ Path	Indirect Effect (ab)	B(SE)	Lower CI	Upper CI
MP → SK → ORTO-15 MP → SK → BOT	−0.1198 ***	−0.0839(ns) 0.0241(ns)	0.0991 *** 0.0222 ***	0.0891 *** 0.0251 ***	0.0101 −0.0029	0.0122 0.0339	−0.0121 −0.0107	0.0355 0.0047
MP → SJ → ORTO-15 MP → SJ → BOT	0.1527 ***	0.4571 *** 0.0875 *	0.0991 *** 0.0222 ***	0.0293(ns) 0.0088(ns)	0.0698 0.0134	0.0177 0.0060	**0.0375** **0.0016**	**0.1070** **0.0254**
MP → CH → ORTO-15 MP → CH → BOT	−0.0542 ***	0.1048(ns) 0.0666(ns)	0.0991 *** 0.0222 ***	0.1048 *** 0.0258 ***	−0.0057 −0.0036	0.0057 0.0023	−0.0177 −0.0087	0.0447 0.0004
MP → IS→ ORTO-15 MP → IS → BOT	0.1464 ****	0.4417 *** 0.0416(ns)	0.0991 *** 0.0222 ***	0.0345(ns) 0.0161(ns)	0.0647 0.0061	0.0190 0.0062	**0.0298** −0.0067	**0.1038** 0.0175
MP → MI → ORTO-15 MP → MI → BOT	−0.0725 ***	0.0572(ns) 0.0671(ns)	0.0991 *** 0.0222 ***	0.1033 *** 0.0271 ***	−0.0041 −0.0049	0.0085 0.0035	−0.0205 −0.0119	0.0129 0.0021
MP → OI → ORTO-15 MP → OI → BOT	0.1163 ***	0.3509 ** −0.0018(ns)	0.0991 *** 0.0222 ***	0.0583 ** 0.0224 **	0.0408 −0.0002	0.0161 0.0054	**0.0095** −0.0116	**0.0723** 0.0099
MP → SC → ORTO-15 MP → SC → BOT	−0.6620 ***	−0.0719 * 0.0043(ns)	0.0991 *** 0.0222 ***	0.0515 * 0.0250 *	0.0476 −0.0028	0.0200 0.0077	**0.0087** −0.0190	**0.0877** 0.0113
AP → SK → ORTO-15 AP → SK → BOT	0.0232(ns)	−0.3013 *** −0.0398(ns)	0.0371(ns) 0.0349 **	0.0441(ns) 0.0358 **	−0.0070 −0.0009	0.0097 0.0016	−0.0267 −0.0044	0.0127 0.0022
AP → SJ → ORTO-15 AP → SJ → BOT	0.1107 ***	0.5731 *** 0.1027 ***	0.0371(ns) 0.0349 **	−0.0263(ns) 0.0235(ns)	0.0635 0.0114	0.0178 0.0048	**0.0313** **0.0036**	**0.1007** **0.0223**
AP → CH → ORTO-15 AP → CH → BOT	0.0508 *	−0.1003(ns) 0.0058(ns)	0.0371(ns) 0.0349 **	0.0422(ns) 0.0346 **	−0.0051 0.0003	0.0060 0.0021	−0.0189 −0.0042	0.0089 0.0048
AP → IS→ ORTO-15 AP → IS → BOT	0.0183(ns)	0.5612 *** 0.0951 **	0.0371(ns) 0.0349 **	0.0269(ns) 0.0331 **	0.0103 0.0017	0.0163 0.0029	−0.0211 −0.0038	0.0435 0.0079
AP → MI → ORTO-15 AP → MI → BOT	0.0573 **	−0.2588 * −0.0284(ns)	0.0371(ns) 0.0349 **	0.0520(ns) 0.0365 **	−0.0148 −0.0016	0.0087 0.0029	**−0.0348** −0.0424	**−0.0008** 0.0034
AP → OI → ORTO-15 AP → OI → BOT	0.0306(ns)	0.5639 *** 0.0738 *	0.0371(ns) 0.0349 **	0.0199(ns) 0.0326 *	0.0172 0.0023	0.0141 0.0023	−0.0102 −0.0012	0.0458 0.0076
AP → SC → ORTO-15 AP → SC → BOT	−0.0283(ns)	−0.1160 *** −0.0170 *	0.0371(ns) 0.0349 **	0.0339(ns) 0.0344 **	0.0033 0.0005	0.0139 0.0022	−0.0241 −0.0041	0.0297 0.0053

Note. MP = maladaptive perfectionism; AP = adaptive perfectionism; ORTO-15 = Orthorexic behaviors; BOT = Bratman Orthorexia Test; SK = Self-Kindness; SJ = Self-Judgment; CH = Common Humanity; IS = Isolation; MI = Mindfulness; OI = Over-Identification; SC = Self-Compassion; ns = not significant; *SE* = standard error; * p < 0.05; ** p < 0.01; *** p < 0.001; Confidence intervals (CI) bounds shown in bold represent mediation paths with statistically significant effects.

## Data Availability

The datasets used during this study are available from the corresponding author.
